# Towards the Simulation of a Realistic Large-Scale Spiking Network on a Desktop Multi-GPU System

**DOI:** 10.3390/bioengineering9100543

**Published:** 2022-10-11

**Authors:** Emanuele Torti, Giordana Florimbi, Arianna Dorici, Giovanni Danese, Francesco Leporati

**Affiliations:** Department of Electrical, Computer and Biomedical Engineering, University of Pavia, 27100 Pavia, Italy

**Keywords:** cerebellar network simulation, graphics processing units, brain modeling, high performance computing

## Abstract

The reproduction of the brain ’sactivity and its functionality is the main goal of modern neuroscience. To this aim, several models have been proposed to describe the activity of single neurons at different levels of detail. Then, single neurons are linked together to build a network, in order to reproduce complex behaviors. In the literature, different network-building rules and models have been described, targeting realistic distributions and connections of the neurons. In particular, the Granular layEr Simulator (GES) performs the granular layer network reconstruction considering biologically realistic rules to connect the neurons. Moreover, it simulates the network considering the Hodgkin–Huxley model. The work proposed in this paper adopts the network reconstruction model of GES and proposes a simulation module based on Leaky Integrate and Fire (LIF) model. This simulator targets the reproduction of the activity of large scale networks, exploiting the GPU technology to reduce the processing times. Experimental results show that a multi-GPU system reduces the simulation of a network with more than 1.8 million neurons from approximately 54 to 13 h.

## 1. Introduction

The modern computational modeling in neuroscience requires to reproduce the activity of large scale and realistic networks in the shortest time. The reproduction of meaningful biological mechanisms requires to include a huge number of elements into the network. Moreover, different models can describe the behavior of single neurons, targeting different levels of details. Among the simplest and most adopted models, the Leaky Integrate and Fire (LIF) features a low computational complexity and can reproduce basic neuronal behaviors such as natural peacemaking and the response to single or multiple input spikes [2]. Thanks to the low computational complexity, the LIF model is the most used to build Spiking Neural Networks (SNNs). However, complex SNNs simulations do not scale linearly when considering large scale problems (i.e., if the number of elements is greater than 100,000) [3]. These limitations have been addressed adopting High Performance Computing (HPC) solutions; in particular, Graphics Processing Units (GPUs) emerged as the ideal solution to simulate large SNNs. In fact, these devices are capable of processing in parallel the neuronal activity of a huge number of cells. One important aspect that can make the GPUs useful in this research field is that they can be hosted in desktop systems as well as in supercomputers.

Recently, Granular layEr Simulator (GES) was proposed [1], which targets multi-GPU systems. This simulator has the advantage of placing the neurons in a three-dimensional space and connecting the elements following biologically realistic rules. As a result, the obtained network features a high level of realism since it reproduces the topology of a real granular layer network. However, the work in [1] describes the single cells activity only through the Hodgkin–Huxley (HH) model, which features a high computational complexity [4]. This is because the HH model describes the cellular activity in a very realistic way and thus requires a set of differential equations and a high number of parameters. Thus, this simulator does not allow to reproduce huge networks on desktop systems, since the GPU memory is limited. In this work, we adopt the same network reconstruction method described in [1] and simulate the cellular activity with LIF models, which are characterized by a differential equation and feature less parameters compared to the HH description. In this way, we extend the functionality of the original GES simulator, allowing the user to choose the preferred model for network simulation. Moreover, since the adopted network is the same as [1], the computational complexity of parallel performance can be directly compared.

Summarizing, the main contributions of this paper are:

- the extension of the GES simulator in order to include the LIF models of the different neurons of the cerebellar granular layer:
- the analysis of the impact of a realistic neuron placements and connections on a multi-GPU simulation of LIF models;
- the direct comparison of the processing times of a network activity simulated in parallel on GPUs adopting HH or LIF models.

Moreover, this work adopts a realistic and innovative approach for placing and connecting the network elements. It is worth noticing that the most of the literature focuses on simulating a huge number of LIF neurons adopting fixed and simple rules to connect the elements (i.e., simple equations or rules that do not represent the real world scenario). On the other hand, most of the works targeting a high biological realism, focuses on the adoption of very complex models such as Hodgkin–Huxley one. The proposed work closes the gap of simulating large scale LIF network considering realistic connection rules. Moreover, the proposed framework extends the existing GES simulator, making possible the simulation of large-scale networks even on desktop systems equipped with GPU boards.

The paper is organized as follows: Section 2 describes the structure of the granular layer network and the LIF models of the different neurons. Section 3 describes the serial algorithm which has been developed for two main purposes: to validate the single neurons behaviors against the literature and to serve as a baseline to evaluate the performance of the parallel algorithm. Moreover, Section 3 details the proposed parallel implementation targeting GPU and multi-GPU systems. The performed experiments and the obtained results are reported in Section 4, together with the discussion and the comparison with the state of the art. Section 5 concludes the paper with some final remarks and hints at future research lines.

## 2. Background

### 2.1. Cerebellar Granular Layer

**Figure 1:**
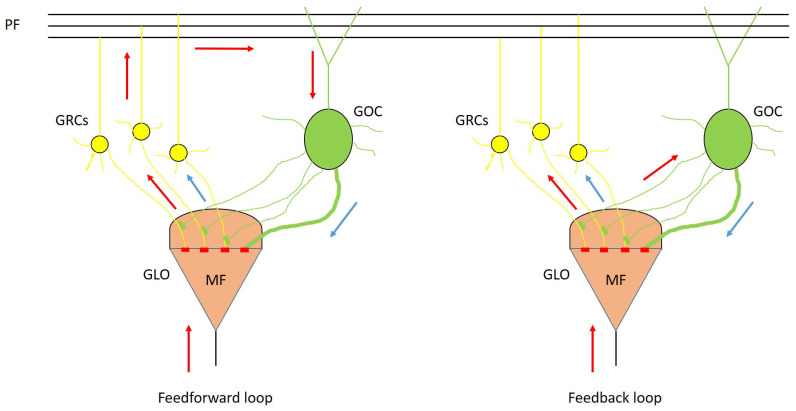
Feedforward and feedback loops. In the first loop, the MF excites the GRCs (yellow spheres), then the signals travel along the parallel fibers and excite the GOC (green sphere) that inhibits the GRCs. In the second loop, the MF excites the GRCs and the GOC, which later inhibits the GRCs. For both the loops, the excitatory signals are shown with red arrows, while the inhibitory ones are represented with blue arrows.

The cerebellar cortex is divided into three parts: the granular, the Purkinje and the molecular layers. Each of these layers includes different types of neurons. The granular layer hosts granule (GRC) and Golgi (GOC) cells. These neurons connect their dendrites and axons in particular structures called glomeruli (GLOs) which are also reached by the mossy fibers (MFs). The connection of these elements constitutes the feedforward and feedback loops, typical of the granular layer [5]. In the feedforward loop, the MFs excite the GRCs and, then, the parallel fibers (PFs) excite the GOCs that inhibit the GRCs. Otherwise, in the feedback loop, the MFs excite the GRCs and GOCs dendrites and these latter inhibit the GRCs. These loops are shown in Figure 1, where the yellow spheres represent the GRC and the green ones the GOC. The excitatory signals are shown with red arrows, and the inhibitory ones by blue arrows. The GRCs, GOCs and MFs are connected through excitatory or inhibitory synapses, depending on the kind of signal exchanged by the neurons.

The elements connectivity in the cerebellar granular layer has been widely studied and, in the literature, a set of rules to connect the neurons has been described [6,7,8,9,10]. The connection rules reported in [1] are obtained by this wide literature review, therefore achieving a very realistic description of the granular layer and also aligned to the state of the art. The network connectivity can be summarized by the following rules:

1. the convergence rate between GLOs and GRCs is 4:1;
2. the GRCs dendrites cannot reach GLOs located at a distance greater than 40 μm;
3. a single GRC cannot send more than one dendrite into the same GLO;
4. each GRC must project its dendrites to four different GLOs;
5. the divergence rule between GLOs and GRCs dendrites is 1:53;
6. the convergence rate between GLOs and the GOCs is 50:1;
7. a GRC is not inhibited twice by the same GOC;
8. each GOC axon can reach and inhibit a maximum of 40 different GLOs;
9. each GOC receives excitatory inputs from about 40 MFs;
10. the convergence rate between the ascending axon and the GOCs is 400:1;
11. the convergence rate between the PFs and the GOCs is 1000:1.

### 2.2. Leaky Integrate and Fire Models

**Figure 2:**
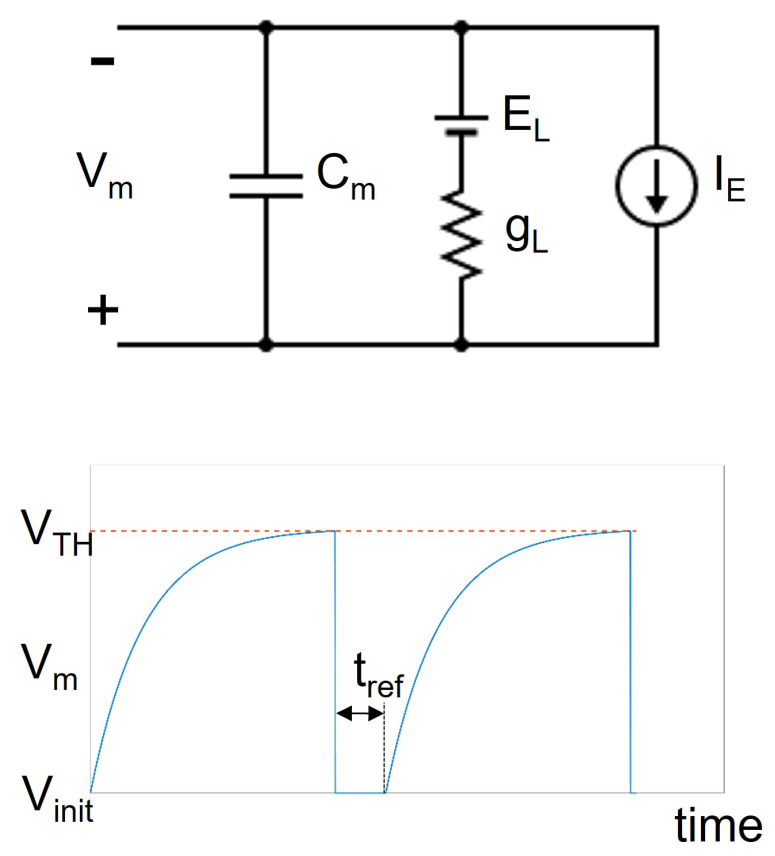
The upper part of the figure shows the circuital model. The lower part represents the voltage of the capacitor $C_{m}$ (blue line) in response to a constant current $I_{E}$. It also shows the voltage reset mechanism: when the $V_{m}$ reaches the threshold $V_{TH}$ (red dashed line), its value is instantly changed to the initial one ($V_{init}$) and it is maintained for a time equal to $t_{ref}$.

The LIF models are among the simplest to describe the neurons activity. They are based on a simple electrical circuit which includes a capacitor $C_{m}$, a conductance $g_{L}$ in series with a voltage generator $E_{L}$ and a current generator $I_{E}$ [11], connected in parallel as shown by Figure 2.

In this circuit, the capacitor $C_{m}$ represents the cellular membrane capacitance, the terms $g_{L}$ and $E_{L}$ are the leakage conductance and voltage, respectively, and the current generator $I_{E}$ denotes the current stimulus. This last term includes the contribution of a constant current used to charge the $C_{m}$ capacitor, the synaptic current generated by the neurons connected to the considered cell and an optional injected current, which is used to externally stimulate the cell in order to validate the model.

The voltage of the capacitor $C_{m}$ is given by Equation (1).

(1) $$C_{m}\frac{dV_{m}}{dt}=I_{E}-g_{L}\left(V_{m}-E_{L}\right)$$

By denoting the leakage current with $I_{L}$ and applying the implicit Euler method, Equation (1) can be rewritten as:

(2) $$\frac{V_{m}\left(t+h\right)-V_{m}\left(t\right)}{h}=\frac{-I_{L}+I_{E}}{C_{m}}$$

where *h* is the integration step. From Equation (2) it is easy to obtain the final expression used to update the membrane voltage of the neuron (Equation (3)).

(3) $$V_{m}\left(t+h\right)=V_{m}\left(t\right)-h\frac{I_{L}+I_{E}}{C_{m}}$$

As written before, the term $I_{E}$ also includes the contribution of the synaptic currents, which can be modeled as:

(4) $$I_{syn}\left(t\right)=g_{syn}\left(t\right)\left(V_{m}\left(t\right)-V_{gen\_syn}\right)$$

where the term $V_{gen\_syn}$ is the equilibrium potential of the considered synapse. It is worth noticing that the synaptic conductance $g_{syn}$ is not constant since it is time dependent. It follows the law expressed by Equation (5).

(5) $$g_{syn}\left(t+h\right)=g_{syn}\left(t\right)e^{-\frac{t}{\tau }}$$

where τ is the synapse time constant. Notice that Equation (5) describes an exponential decay from the initial value of $g_{syn}$ to 0. Moreover, the value of $g_{syn}$ is incremented by a fixed quantity *w* every time a spike signal generated from a neuron reaches the considered synapse. Thus, the incoming spikes to the synapses instantly change the value of $g_{syn}$, triggering the exponential decay mechanism (Equation (5)).

To reproduce the firing rate of a neuron, Equation (1) is extended with a threshold mechanism, in order to reset the $V_{m}$ potential to an initial value, denoted with $V_{init}$. The threshold mechanism is described by Equation (6).

(6) $$V_{m}\left(t+h\right)=\left\{\begin{array}{cc} Equation\;(3) & \mathrm{if}\;V_{m}\left(t\right)<V_{TH}\\ V_{init} & \mathrm{if}\;V_{m}\left(t\right)\geq V_{TH} \end{array}\right.$$

Finally, every time the voltage $V_{m}$ reached the threshold value $V_{TH}$, $V_{m}$ is reset to the initial value $V_{init}$, the value is maintained constant for a time $t_{ref}$ which is called *refractory period*.

The lower part of Figure 2 shows the $V_{m}$ voltage waveform after a constant current injection (solid blue line). It is worth noticing that, after reaching the value $V_{TH}$ highlighted by the dashed red line, the $V_{m}$ is reset to the initial value and keep constant for the refractory period ($t_{ref}$). After the refractory period, the model starts to evaluate again Equation (3).

### 2.3. Methods for Numerical Integration of Ordinary Differential Equations

The system shown by Equation (6) is based on the ordinary differential equation (ODE) of Equation (1). Several methods have been proposed the solve ODEs. The choice of the numerical integration method has a great influence on the computational time and accuracy [12]. Among the different ODEs solvers proposed in the literature, the most used are the forward Euler, Heun, Adams–Basforth, Runge–Kutta methods [13]. All these methods perform interpolation at each integration step to approximate the derivative at a discrete subset of time points.

It is worth noticing that the LIF model adopts first order differential equation and that a large-scale network includes hundreds of thousands of neurons. Moreover, this work targets the simulation of long periods of cellular activities. Thus, the differential equations should be solved a huge number of times for each neuron. Therefore, the proposed work adopts the Euler implicit method to solve the differential equations, being the one that ensures a low computational complexity and an error which is negligible for the considered application. Note that the Euler method is used in other applications adopting High Performance Computing architecture to speed up the computation [14,15,16].

## 3. Integration of the LIF Models into the GES Simulator

### 3.1. Validation of the GRC and GOC Simulations

The LIF models described in the previous section can reproduce the behavior of the GRC and GOC cells, which are the neurons included in the granular layer. It is worth noticing that these neurons can be described by Equations (3) and (6), changing the parameters $g_{L}$, $E_{L}$, $I_{E}$, $C_{m}$, $V_{TH}$, $V_{init}$ and $t_{ref}$. The parameters used to describe the GRC and GOC cells have been taken from the NeuroElectro Database [17]. The parameters values used for each cell type are reported in Table 1.

Considering the synapses, the excitatory and inhibitory ones are characterized by the parameters $V_{gen\_syn}$, τ, *w* and the initial value of $g_{syn}$. Again, the parameters values have been taken from the NeuroElectro Database [17] and reported in Table 2.

The analysis of the values reported in Table 1 and Table 2 allows to compare the behaviors of the GRC and GOC. It is important to notice that the parameter $I_{E}$ is equal to zero only for the GRC. This means that, in normal conditions, the GRC maintains its resting potential $V_{init}$. On the other hand, the GOC is characterized by a positive value of $I_{E}$. This means that this cell is characterized by a natural peacemaking, i.e., the potential varies as a periodical signal which grows from $V_{init}$ to $V_{TH}$ and then is reset to $V_{init}$. Moreover, the role of the synapses is to instantly change the value of the potential when they receive a spike. In particular, the excitatory synapse increases the potential, while the inhibitory one decreases the value.

**Figure 3:**
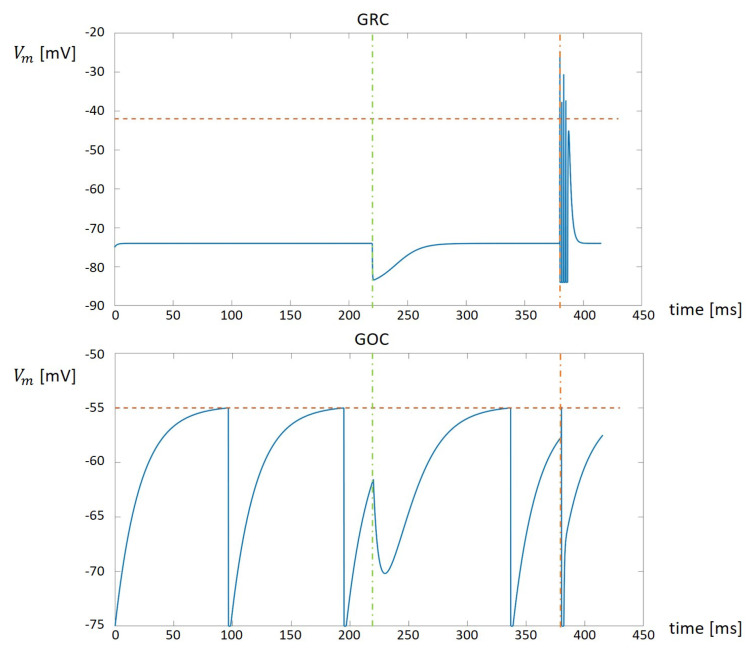
The results produced by the single cells C simulators.

In order to integrate the LIF models of the GRC and GOC into the GES simulator, authors developed a serial C code to solve Equation (6). The results obtained by these single cells simulators have been compared with the one reported in the NeuroElectro Database, in order to validate the developed serial C codes. Examples of the output produced by these simulators are reported in Figure 3.

The top part of Figure 3 shows the result produced by the GRC serial simulator. It is worth noticing that, at the beginning, the potential is fixed to the $V_{init}$ value. When an inhibitory synapse is activated by a spike (vertical green dashed line) the potential instantly diminishes and then grows back to the $V_{init}$ value. On the other hand, when an excitatory synapse is activated by a spike (vertical red dashed line) the potential is characterized by the presence of spikes. When the excitatory effect ends, the potential comes back again to the $V_{init}$ value. The bottom part of Figure 3 shows the GOC activity. At the beginning, the GOC shows its natural peacemaking. When the inhibitory synapse is activated (vertical green dashed line), the potential is instantly decreased and, then, the cells continues with its natural peacemaking. Considering the excitatory input (vertical red dashed line), it instantly increments the potential. In the case shown in Figure 3, the excitatory input increases the potential to a value greater then the threshold. The value is then reset to $V_{init}$ and, after the refractory time, the cells restart with the natural peacemaking.

In order to integrate these two serial simulators into the GES software, authors developed a serial simulator of the cerebellar granular layer, exploiting the cells placing and connection module of the GES simulator. This module produces as output a set of connection matrices that are used to route the signals to the cells. For each GRC and GOC, in each synapse, a suitable array is allocated. Thus, these arrays are used to store the spike times related to a synapse and it is removed from the array when a spike is processed by the synapse. Concerning the GRCs, the excitatory inputs are generated by the MF, while for the GOCs they are generated from the MFs or from the GRCs. On the other hand, the inhibition signals are generated by the GOCs for the GRCs. The MFs are initialized using the spike train generator already described in [1]. The arrays storing the spikes queues not generated by the MFs change their content during the simulation depending on which neurons generate a spike.

**Figure 4:**
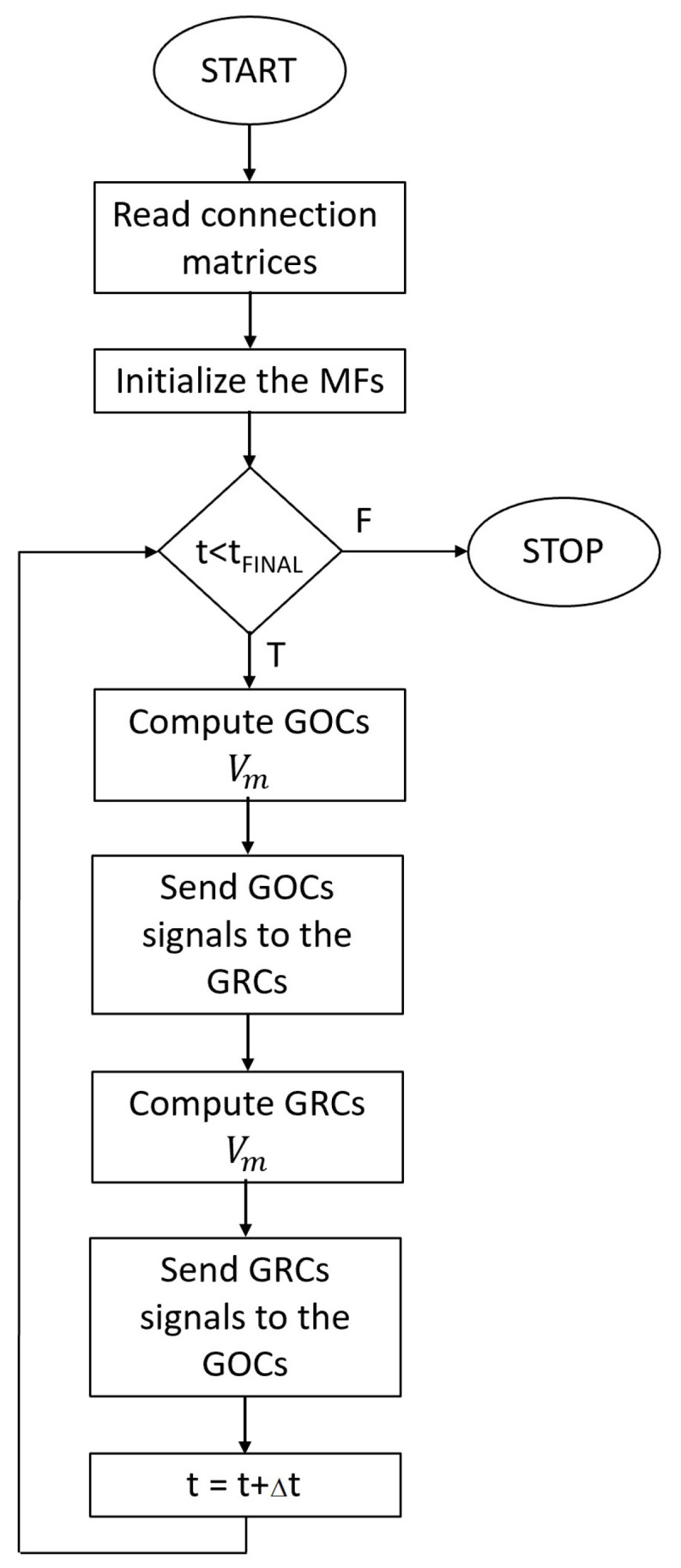
The flowchart of the serial network simulator.

The flowchart of the serial simulator is shown in Figure 4. After reading the connection matrices and initializing the MFs, the simulator starts to evaluate the main *for* loop related to the time. For each time step, the cellular activity of the GOCs is computed (i.e., $V_{m}$ evaluated). If the potential of each GOC reaches the value $V_{TH}$, this means that this cell has generated a spike. Therefore, the time stamp of the spike is computed by summing to actual time *t* a pseudo-random value related to the propagation time from the *i*-th GOC to the connected cells. This time stamp is then stored in the inhibitory spike queues of all the GRCs connected to the *i*-th GOC.

At this point, the GRCs potentials have been updated; a spike evaluation mechanism similar to the one previously described is implemented in order to store the time stamps into the GOCs excitatory queues. The main loop is repeated until the actual simulated time *t* is lower than the final time $t_{fin}$.

### 3.2. Parallel Implementation of the Granular Layer Network

**Figure 5:**
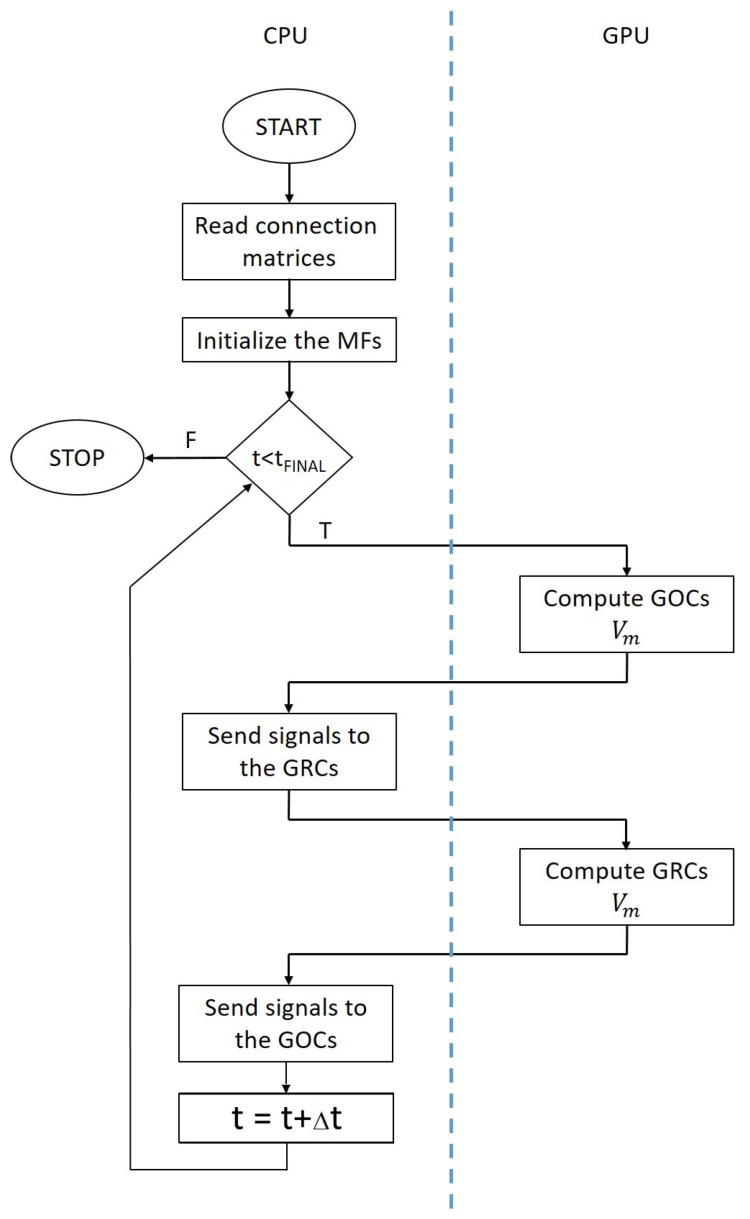
The flowchart of the GPU-based network simulator.

By analyzing the flowchart reported in Figure 4, the computational complexity of the simulation is related to the main *for* loop iterating over the time steps. Moreover, inside this loop, the evaluation of the GOCs and GRCs activities is the most time consuming operations because the simulator should solve Equation (6) for each cell. Finally, the signal routing to the suitable queues should be performed serially, since more than one cell can write to the same queue at the same iteration *t*, but the stored time stamp must be in ascending order to correctly evaluate the spikes. Thus, we developed a CUDA-based algorithm to reproduce in parallel the activity of the GOCs and GRCs. The flowchart reported in Figure 5 highlights the tasks performed by the CPU in serial and the parallel elaboration carried out by the GPU. Moreover, data transfers are represented by arrows passing through the blue dashed line.

As for the serial network simulator, the first tasks are related to the acquisition of the connection matrices and the generation of the spike queues related to the MFs. Then, the main *for* loop can begin. The first task checks if each queue related to each GOC contains a spike at the actual simulation time. If this condition is true, a flag is set into a suitable array in the *i*-th position to indicate that the GOC connected to the *i*-th synapse is receiving a stimulus. Thus, after evaluating each queue, an array of flags is transferred to the GPU memory. Then, a number of parallel threads equal to the number of GOC is used by the GPU to carry out the cellular activity simulation. Thus, each GPU thread solves Equation (6), considering the presence of a spike in each synapse by checking the flags array portion related to the considered neuron. It is worth noticing that the parameters and variables values needed to solve Equation (3) are stored in suitable arrays allocated on the GPU memory before the main loop. On the other hand, the constant parameters are defined as macro and then substituted with the corresponding value at compilation time. If the *i*-th GOC has produced a spike at the current time (i.e., its potential is equal or grater than $V_{TH}$), a flag is set in the *i*-th position of an array, then is transferred back to the CPU memory. The information stored in this array are used by the CPU to set flags into the GRCs inhibitory queues. In particular, if the *i*-th GOC is connected to the *j*-th GRC and this GOC produces a spike, the flag array related to the inhibitory queue of the *j*-th GRC is set true. Moreover, the spike queues related to the MFs are checked in order to correctly set the flag array related to the excitatory synapses. These flag arrays are then transferred to the GPU memory, as for the flag array related to the GOC. On the GPU, a set of threads equal to the number of GOCs is created to evaluate in parallel the activity of these neurons. After updating the GRC potential value, each thread checks if the assigned cell produced a spike at the current time. Again, the memory addresses of the flag array related to the GRC that generated a spike are set to the true value. This array is transferred back to the CPU memory, where it is used, together with the MFs queues to generate the signals for the GOC of the next iteration of the main loop.

This simulation framework can be generalized to exploit multi-GPU systems by using CUDA *streams*, which allow asynchronous data transfers and GPU kernel execution. In particular, a stream is associated to each GPU of the system. Thus, each GPU elaborates data independently from the other devices. This strategy can be applied only to the GOCs and GRCs activity evaluation, since, as explained before, the queues should be serially managed. Therefore, a synchronization barrier is placed before each serial code block. Notice that all the other GPU related operations (memory transfers and kernels executions) are managed through CUDA *streams*; therefore, the data transfers and kernel executions of different devices are overlapped.

In general, considering a system equipped with *N* GPUs, each device performs the activity simulation of an equal number of neurons. If N_GOCs denotes the total number of GOCs and N_GRCs is the total number of GRCs, each GPU elaborates $\frac{N\_GOCs}{N}$ and $\frac{N\_GRCs}{N}$ neurons.

## 4. Experimental Results and Discussion

### 4.1. Performed Experiments

The granular layer network based on LIF models has been validated exploiting the GES neuron placement and connection module. We used this module to generate three networks configurations with different sizes, in order to characterize the computational complexity and the scalability of these models. The main features of these networks are reported in Table 3.

The physical size of the networks is chosen in order to consider large scale problems. In these specific cases, *Network1*, *Network2* and *Network3* include about 116,000, 465,000 and 1,850,000 elements. These networks dimensions were selected with a group of neuroscientists belonging to the same university.

As explained in Section 2.1, the MFs provide the input to the network. Therefore, the simulation of the MFs activity following suitable protocols is mandatory to carry out a realistic simulation of the cerebellar granular layer. In [1] four different protocols are used both to simulate the network and to characterize the system performance. The four protocols are summarized in Table 4.

*Prot1* is a typical protocol used in neuroscience, since it reproduces the normal background activity. In this case, the MFs receives a 1 Hz signal, i.e., the MFs are stimulated with a spike every second. It is worth noticing that the spike times are different for each MF, even if their frequency is the same. *Prot2* represents another realistic neuroscience stimulus which is called *burst*. In this case, the MF is stimulated with a 100 Hz signal for a very short time. In this protocol, only 10% of the MFs are stimulated. The third protocol (*Prot3*) is a combination of the previous two stimuli. In fact, all the MFs receive both the background and the burst. From the neuroscience point of view, this protocol is realistic and it is used only to perform a stress test on the network and to characterize the performance of the system. Finally, *Prot4* is a realistic stimulus in which all the MFs receive a 1 Hz background and 1% of the MFs also receives a 100 Hz burst.

Another important aspect to consider is the physical time to simulate. In order to compare our results with the work in [1], the simulations consider 1, 3 and 10 s of activity. Moreover, simulations of 30 and 60 s have also been carried out to best characterize the performance of the proposed parallel simulator.

The simulations have been performed on a system equipped with an Intel i9 9900X processor with 128 GB of RAM. This CPU is connected to two NVIDIA RTX 2080 GPU featuring 4608 CUDA cores working at 1.35 GHz and 16 GB of RAM.

### 4.2. Processing Times

All the protocols shown in Table 4 have been used to stimulate the three networks to simulate 1, 3, 10, 30 and 60 s of activity. The processing time of the serial simulations are reported in Table 5. As expected, for each stimulation protocol, the processing times scale up with the network sizes and the duration of the simulated cellular activity. To evaluate how the different protocols impact on processing times, considerations about the synaptic activities should be carried out. *Prot1* stimulates the synapses with a background signal at the frequency of 1 Hz. This means that all the synapses linked to a specific MF receive a spike signal every second. Thus, for the considered simulated times, each MF receives a number of spikes ranging from approximately 1 to 60. It should be noticed that the number of spikes is not fixed since the spikes queues are generated adopting a Poisson distribution. This protocol serves as base for the other stimulations; therefore, the other protocols require to evaluate the synapses activities more time than in *Prot1*. In particular, *Prot3* is the one generating the greatest number of spikes since it includes the background activities of *Prot1* and a burst of 100 Hz for 50 ms used for the stimulation of all the MFs. This burst results in about five to seven additional spikes that are evaluated by the MFs. *Prot2* and *Prot4* require to evaluate a number of spikes which is lower than *Prot3*, resulting in lower processing times. Summarizing, for each network, *Prot3* requires the longest processing times. This is because it evaluates the synapses contributions more times than the other protocols, since all the MFs are stimulated both by a background and a burst. Notice that there is a 4× factor between the number of elements included in each network. Considering the same protocol and the same simulated time, this factor can also be identified in the processing times. Taking a single network, for each protocol, the simulation times linearly scales up with the duration of the simulated cellular activity. Therefore, the processing times linearly grows up both with the network size (i.e., the number of elements to simulate) and the duration of the simulated cellular activity.

Finally, the serial processing times range from 4 h (*Network1*) to 60 h and 40 min (*Network3*), which clearly demonstrates the need of parallel processing to efficiently simulate large scale networks for long time periods.

The processing times of the parallel code are summarized in Table 6, Table 7 and Table 8. As a general comment, the parallel algorithm achieves a speed-up ranging from about 1.50× to about 7.00×. The lowest speed-up is related to the *Network1* when stimulated with *Prot3* to simulate 1 s of cellular activity. This is due to two main factors. The former is the low number to elements to simulate (less than 110,000), while the latter is the high number of spikes generated by the stimulation protocol. In fact, all the MFs generate a burst during 1 s of simulated cellular activity. This means that the probability that the bursts of different MFs are generated at similar time steps is high. Thus, all the cells reach a high number of stimuli that are evaluated at the same time. Notice that the evaluation of a spike to calculate the synaptic current is based on *if* statements, which cause branches divergence degrading the GPU performance.

This limit is overcome if the number of elements included in the network augments. In fact, the speed-ups for *Network2* and *Network3* when considering *Prot3* are not negligible.

**Figure 6:**
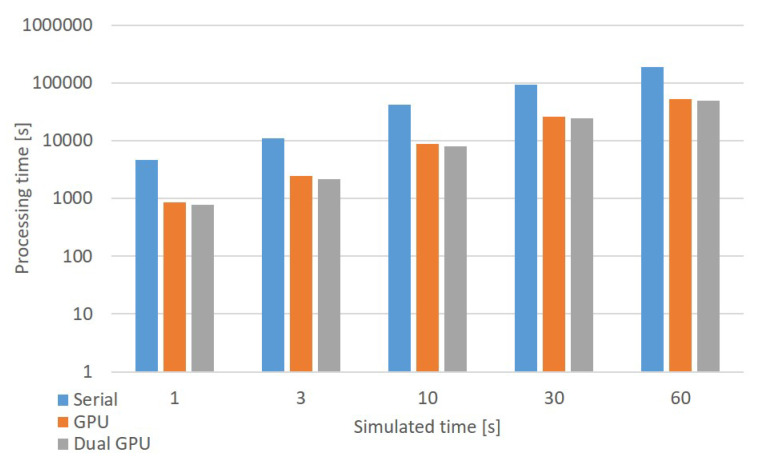
Chart of the processing time of the *Network3* when stimulated with *Prot4*. The processing time is represented in logarithmic scale.

All the networks show speed-ups ranging from about 3.50× to about 7.00× when considering *Prot1*, *Prot2* and *Prot4*. These three protocols represent situations that are of interest for neuroscientists. Thus, the proposed parallel algorithm integrated into the GES simulator offers an interesting solution to simulate large scale networks even on a desktop PC. These considerations are confirmed by Figure 6, which shows the performance of the systems considering *Network3* when stimulated with *Prot4*, which is a typical protocol considered by neuroscientists.

### 4.3. Memory Occupancy Analysis

As described in Section 3.2, the parallel algorithm describes the GOCs and the GRCs adopting LIF models with the parameters shown in Table 1 and Table 2. Thus, each element features seven parameters for the cellular activity and four for each synaptic receptor. The model of the granular layer of the GES simulator considers four excitatory and four inhibitory receptors for the GRC, and two excitatory and one inhibitory receptors for the GOC. Therefore, considering the IEEE754 single precision floating point format, each GRC requires 156 B while each GOC needs 76 B. The GPU memory should also store the flag arrays related to the synapse, as described in Section 3.2. Thus, there is an additional memory space required of 32 B and 12 B for each GRC and GOC, respectively.

Summarizing, the amount of GPU memory to simulate a generic network is given by Equation (7):

(7) $$RAM_{GPU}=88\times N\_GOC+188\times N\_GRC$$

where N_GOC and N_GRC indicate the number of GOC and of GRC, respectively.

Applying Equation (7) to the networks described in Table 3, it is possible to compute the memory occupancy of *Network1*, *Network2* and *Network3*. Thus, *Network1* requires 19.77 MB, *Network2* takes 77.53 MB and *Network3* occupies 310.14 MB. These results clearly demonstrate that the proposed parallel algorithm can simulate large scale networks even on mid-range GPUs, since it does not requires an excessive amount of memory. It is important to highlight that a mid-range device typically features a lower number of cores than the GPU considered in this study. This will cause an increase in the simulation times, but the processing will be completed even on mid-range boards. On the other hand, if the algorithm is ported on a system with multiple GPUs, the size of the network can be further increased.

### 4.4. Comparison with the State of the Art

In the literature, the parallel simulation of the cerebellar granular layer has been explored by several researchers. In [18,19], Naveros et al. described a hybrid CPU-GPU simulator which integrates time-driven and event-driven techniques. The system was capable to simulate 50000 neurons in real-time. However, the authors did not adopt a realistic placing and connection method, since the neurons links are randomly generated. Moreover, the cellular activity was simulated adopting look up tables rather than directly solving differential equations.

Yamazaki et al. [20] reproduced the cat cerebellum network containing more than a billion spiking neurons. They simulated 1 s of cellular activity in real time exploiting 1280 PEZY-SC processors and adopting LIF models. It is worth noticing that this real time compliant simulation is performed by the Shobun supercomputer and not by a desktop system. This is a crucial difference with the work proposed in this paper, since it aims at providing a parallel large scale networks simulator which can run on desktop PCs. Finally, the reconstruction of the cerebellum is based on the PEZY-SC architecture and not on the physiological structure of the brain.

NeuroConstruct [21] is a tool to build and visualize neuronal networks in a 3D space. The network design reproduces very realistic and complex neuron morphologies exploiting the Hodgkin and Huxley model. The system was validated with simulations up to only 5000 neurons on a single-processor machine that takes 1–2 h for 4 s of activity. Even if the morphology is very detailed, the tool does not allow to build large scale networks with sizes comparable to the one adopter in this paper. Concerning the computational time, a direct comparison would not be fair since NeuroConstruct adopts a model featuring a higher computational complexity than the LIF one.

In [22] the LIF neurons were implemented on the same GPU considered by the proposed work. Authors simulated 1 million neurons taking 70 s of processing time per second of biological activity. However, this work considers a highly connected and balanced cortical microcircuit model, which is very different from the cerebellar granular layer. Thus, a direct comparison would not be fair.

CARLsim 4 [23] is an open source library to build and simulate large scale network on heterogeneous clusters. This library has been validated on a random spiking network with 80% excitatory neurons and 20% inhibitory cells. Again, this work does not consider a network with realistic physiological constraints.

Realistic simulation of large scale networks have been addressed also in [24] where a network with 1,000,000 neurons is considered. The work claims to reach a quasi real time simulation adopting the LIF or the Izhikevich models. However, the connections between neurons have been managed only considering a synapse to neuron ratio of 100. Again, a direct comparison would not be fair since the connection rules are very simple with respect to the GES ones.

To the best of authors’ knowledge, the works adopting neurons displacement and connections with a realism nearly comparable with the GES approach are [25,26]. Both these works focused on the reconstruction and simulation of a network with size 400×400×900 μm${}^{3}$ with less than 100,000 neurons. The first work did not report details about the system and the processing times, while the latter is focused on the communication profiling of the simulation on the SpiNNaker neuromorphic hardware. Moreover, it is important to notice once more that these two works were implemented on highly parallel HPC systems rather than desktop-based solution.

The SpiNNaker system is also adopted in [27,28] to achieve real-time simulation of LIF neurons-based networks including 80,000 neurons. It is worth noticing that this result has been achieved employing a system equipped with 1 million of ARM cores as main processing units working at 200 MHz. Thus, the architecture of the SpiNNaker system is very different compared to the GPU structure. One more time, a direct performance comparison would not be fair.

An interesting work addressing the importance of realistic modelling of neurons placement is [29]. This work considers the hippocampus. The network simulations were carried out in parallel on 5 nodes and 36 processors of the Piz-Daint supercomputer available at the Swiss National Supercomputer Center. The cellular activity simulated time is 35 ms. No other information is given about the time taken by the supercomputer to simulate the network. Again, this work is not directly comparable with our research, but highlights the importance of realistic rules to connect and place the neurons.

Authors of [30] reproduced the activity of 32 mm${}^{2}$ of a macaque cortex on 32 Tesla V100 GPUs. The parallel implementation achieved a speed-up of about 3 compared to the NEST simulator baseline. It is important to highlight that the speed-up is lower compared to the one achieved by our parallel algorithm. However, the paper focuses only on the spike delivery algorithm, while the neurons simulation is automatically managed by NEST.

## 5. Conclusions

In this paper, the development of a single and multi-GPU simulation module adopting LIF neurons is presented. These simulators adopt a realistic neurons placements and connections approach and target large scale networks.

A parallel approach targeting multi-GPU desktop system has been described. The simulator has been validated on three networks of different sizes, considering four protocols and several durations of cellular activity to reproduce. The considered protocols have been designed for two main purposes. The first is to realistically reproduce the inputs given to a neuronal network, while the latter is to perform a stress test to evaluate the processing performance.

Experimental results demonstrate the effectiveness of the single and multi-GPU approach, making possible the simulation on large scale networks on desktop PCs in acceptable times without the need of expensive HPC systems. In particular, the dual-GPU system achieves a maximum speed-up of about 7 compared to serial processing.

Future works will consider the inclusion of the molecular and Purkinje layers in order to reproduce the activity of the whole cerebellum. Finally, the proposed work paves the way to the inclusion of large-scale networks in neurorobotic platforms. Indeed, neurorobots platforms simulations such as the one described in [31] adopt LIF models. We proposed a parallel implementation which is not machine dependent, but it is compatible with any CUDA-enabled devices. Thus, the proposed parallel implementation can be used also in low power and portable GPUs. 

## Figures and Tables

**Table 1:** LIF models parameters for the GRC and GOC cells.

Parameter	GRC	GOC
$g_{L}$	1.5 nS	3.6 nS
$E_{L}$	−74 mV	−65 mV
$I_{E}$	0 pA	36.75 pA
$C_{m}$	3 pF	76 pF
$V_{TH}$	−42 mV	−55 mV
$V_{init}$	−84 mV	−75 mV
$t_{ref}$	1.5 ms	2 ms

**Table 2:** Parameters for the excitatory and inhibitory synapses.

Parameter	Excitatory Synapse	Inhibitory Synapse
$V_{gen\_syn}$	0 mV	−85 mV
τ	0.5 ms	10 ms
*w*	20 nS	−10 nS
$g_{syn}$	0 nS	0 nS

**Table 3:** Features of the simulated granular layer networks.

Network ID	Physical Size [μm${}^{3}$]	# of GOCs	# of GRCs	# of GLOs
*Network1 *	300×75×1200	243	108,000	8099
*Network2*	600×150×1200	972	432,000	32,399
*Network3*	1200×300×1200	3888	1,728,000	129,599

**Table 4:** Features of the simulated granular layer networks.

Protocol ID	Stimulus Type	Description
*Prot1*	Background	All the MFs receive a background signal of 1 Hz
*Prot2*	Burst	10% of the MFs receive a 100 Hz burst for 50 ms
*Prot3*	Background + Burst	All the MFs receive both the 1Hz background and the 100 Hz burst
*Prot4*	Background + Burst	All the MFs receive the 1Hz background and 1% of the MFs receive the 100 Hz burst

**Table 5:** Processing time for the serial simulations.

Protocol ID	Simulated Time	*Network1* [s]	*Network2* [s]	*Network3* [s]
*Prot1*	1 s	236.56	987.64	4125.66
	3 s	709.50	2614.12	9708.80
	10 s	2337.08	9896.63	41,761.12
	30 s	7133.05	25,676.48	92,690.36
	60 s	14,386.50	51,586.48	188,804.76
*Prot2*	1 s	226.03	973.94	4164.44
	3 s	694.90	2853.13	11,754.36
	10 s	2298.36	9391.82	39,060.12
	30 s	6909.95	28,040.60	94,235.90
	60 s	14,508.90	55,835.09	192,035.60
*Prot3*	1 s	274.91	1012.14	4244.40
	3 s	751.48	3260.60	11,514.90
	10 s	2404.31	10,201.35	47,243.92
	30 s	7198.80	29,248.08	111,297.12
	60 s	14,365.90	59,023.66	218,487.75
*Prot4*	1 s	238.82	959.92	3674.35
	3 s	711.92	3031.86	10,924.85
	10 s	2366.03	9926.13	41,630.17
	30 s	7093.74	28,980.29	93,543.60
	60 s	14,017.80	57,575.27	189,987.45

**Table 6:** Processing time for the network *Network1*. The speed-up is indicated between brackets.

Protocol ID	Simulated Time	Single GPU [s]	Dual GPU [s]
*Prot1*	1 s	47.56 (4.97×)	43.22 (5.47×)
	3 s	148.38 (4.78×)	136.57 (5.19×)
	10 s	520.62 (4.49×)	460.42 (5.07×)
	30 s	1541.12 (4.63×)	1397.52 (5.10×)
	60 s	3104.64 (4.63×)	2968.01 (4.85×)
*Prot2*	1 s	54.62 (4.14x×)	53.04 (4.26×)
	3 s	153.66 (4.52×)	141.18 (4.92×)
	10 s	480.15 (4.79×)	442.61 (5.19×)
	30 s	1419.04 (4.87×)	1321.09 (5.23×)
	60 s	2834.40 (5.12×)	2790.60 (5.19×)
*Prot3*	1 s	179.46 (1.53×)	186.89 (1.47×)
	3 s	288.97 (2.60×)	272.44 (2.76×)
	10 s	645.57 (3.72×)	593.48 (4.05×)
	30 s	1680.93 (4.28×)	1520.23 (4.74×)
	60 s	3328.86 (4.32×)	3255.98 (4.41×)
*Prot4*	1 s	50.84 (4.70×)	46.11 (5.18×)
	3 s	152.96 (4.65×)	139.16 (5.12×)
	10 s	512.87 (4.61×)	464.15 (5.10×)
	30 s	1557.26 (4.55×)	1422.61 (4.99×)
	60 s	3085.59 (4.54×)	2942.33 (4.76×)

**Table 7:** Processing time for the network *Network2*. The speed-up is indicated between brackets.

Protocol ID	Simulated Time	Single GPU [s]	Dual GPU [s]
*Prot1*	1 s	189.82 (5.20×)	174.01 (5.68×)
	3 s	603.00 (4.34×)	560.75 (4.66×)
	10 s	2142.19 (4.62×)	1966.22 (5.03×)
	30 s	6470.93 (3.97×)	6000.40 (4.28×)
	60 s	12,850.37 (4.01×)	11,745.08 (4.39×)
*Prot2*	1 s	276.86 (3.52×)	272.24 (3.58×)
	3 s	637.19 (4.48×)	596.66 (4.78×)
	10 s	1910.17 (4.92×)	1766.67 (5.32×)
	30 s	5509.73 (5.09×)	5089.96 (5.51×)
	60 s	10,960.07 (5.90×)	10,072.78 (5.54×)
*Prot3*	1 s	284.75 (3.55×)	277.40 (3.65×)
	3 s	645.32 (5.05×)	599.73 (5.44×)
	10 s	2438.64 (4.18×)	1985.40 (5.14×)
	30 s	6436.90 (4.54×)	6132.07 (4.77×)
	60 s	12,904.21 (4.57×)	12,649.72 (4.67×)
*Prot4*	1 s	208.02 (4.61×)	191.69 (5.01×)
	3 s	613.90 (4.94×)	564.34 (5.37×)
	10 s	2117.95 (4.69×)	2249.99 (4.41×)
	30 s	6375.53 (4.55×)	5933.43 (4.88×)
	60 s	12,755.98 (4.51×)	12,077.51 (4.77×)

**Table 8:** Processing time for the network *Network3*. The speed-up is indicated between brackets.

Protocol ID	Simulated Time	Single GPU [s]	Dual GPU [s]
*Prot1*	1 s	759.61 (5.43×)	700.59 (5.89×)
	3 s	2450.53 (3.96×)	2299.41 (4.22×)
	10 s	8832.45 (4.73×)	8396.73 (4.97×)
	30 s	27,154.46 (3.41×)	25,636.35 (3.61×)
	60 s	51,168.78 (3.69×)	46,177.91 (4.09×)
*Prot2*	1 s	703.36 (5.92×)	597.33 (6.97×)
	3 s	2642.27 (4.45×)	2421.63 (4.85×)
	10 s	7499.19 (5.21×)	7051.63 (5.54×)
	30 s	21,229.72 (4.44×)	18,810.85 (5.01×)
	60 s	42,380.45 (4.53×)	34,897.29 (5.50×)
*Prot3*	1 s	997.43 (4.25×)	889.35 (4.77×)
	3 s	2933.45 (3.92×)	2644.90 (4.35×)
	10 s	9131.19 (5.17×)	8835.59 (5.35×)
	30 s	29,297.30 (3.80×)	28,547.15 (3.90×)
	60 s	53,235.23 (4.10×)	51,783.19 (4.22×)
*Prot4*	1 s	851.15 (4.32×)	775.90 (4.74×)
	3 s	2434.87 (4.49×)	2187.59 (4.99×)
	10 s	8746.29 (4.76×)	7906.94 (5.26×)
	30 s	26,101.86 (3.58×)	24,347.18 (3.84×)
	60 s	52,433.85 (3.62×)	49,575.08 (3.83×)

## Data Availability

Data sharing not applicable.
